# Oral versus intravenous antibiotic treatment for bone and joint infections (OVIVA): study protocol for a randomised controlled trial

**DOI:** 10.1186/s13063-015-1098-y

**Published:** 2015-12-21

**Authors:** Ho Kwong Li, Matthew Scarborough, Rhea Zambellas, Cushla Cooper, Ines Rombach, A. Sarah Walker, Benjamin A. Lipsky, Andrew Briggs, Andrew Seaton, Bridget Atkins, Andrew Woodhouse, Anthony Berendt, Ivor Byren, Brian Angus, Hemant Pandit, David Stubbs, Martin McNally, Guy Thwaites, Philip Bejon

**Affiliations:** Nuffield Department of Medicine, University of Oxford, Oxford, UK; Oxford University Hospitals NHS Trust, Oxford, UK; Nuffield Department of Orthopaedics, Rheumatology and Musculoskeletal Science, University of Oxford, Oxford, UK; MRC Clinical Trials Unit, University College London, London, UK; Division of Medical Sciences, University of Oxford, Oxford, UK; Health Economics and Health Technology Assessment, University of Glasgow, Glasgow, UK; Gartnavel General Hospital, NHS Greater Glasgow and Clyde, Glasgow, UK; Heart of England NHS Foundation Trust, Birmingham, UK; Oxford University Clinical Research Unit, Wellcome Trust, Ho Chi Minh, Vietnam; Kenya Medical Research Institute, Wellcome Trust, Kilifi, Kenya

**Keywords:** Bone and joint infection, Oral, Intravenous, Antibiotic, Non-inferiority, Treatment failure

## Abstract

**Background:**

Bone and joint infection in adults arises most commonly as a complication of joint replacement surgery, fracture fixation and diabetic foot infection. The associated morbidity can be devastating to patients and costs the National Health Service an estimated £20,000 to £40,000 per patient.

Current standard of care in most UK centres includes a prolonged course (4–6 weeks) of intravenous antibiotics supported, if available, by an outpatient parenteral antibiotic therapy service. Intravenous therapy carries with it substantial risks and inconvenience to patients, and the antibiotic-related costs are approximately ten times that of oral therapy. Despite this, there is no evidence to suggest that oral therapy results in inferior outcomes.

We hypothesise that, by selecting oral agents with high bioavailability, good tissue penetration and activity against the known or likely pathogens, key outcomes in patients managed primarily with oral therapy are non-inferior to those in patients treated by intravenous therapy.

**Methods:**

The OVIVA trial is a parallel group, randomised (1:1), un-blinded, non-inferiority trial conducted in thirty hospitals across the UK. Eligible participants are adults (>18 years) with a clinical syndrome consistent with a bone, joint or metalware-associated infection who have received ≤7 days of intravenous antibiotic therapy from the date of definitive surgery (or the start of planned curative therapy in patients treated without surgical intervention). Participants are randomised to receive either oral or intravenous antibiotics, selected by a specialist infection physician, for the first 6 weeks of therapy. The primary outcome measure is definite treatment failure within one year of randomisation, as assessed by a blinded endpoint committee, according to pre-defined microbiological, histological and clinical criteria. Enrolling 1,050 subjects will provide 90 % power to demonstrate non-inferiority, defined as less than 7.5 % absolute increase in treatment failure rate in patients randomised to oral therapy as compared to intravenous therapy (one-sided alpha of 0.05).

**Discussion:**

If our results demonstrate non-inferiority of orally administered antibiotic therapy, this trial is likely to facilitate a dramatically improved patient experience and alleviate a substantial financial burden on healthcare services.

**Trial registration:**

ISRCTN91566927 - 14/02/2013

**Electronic supplementary material:**

The online version of this article (doi:10.1186/s13063-015-1098-y) contains supplementary material, which is available to authorized users.

## Background

Infections involving bone and joint have become increasingly common. In the National Health Service (NHS) in the UK, approximately 250,000 orthopaedic operations are performed annually including 160,000 hip and knee replacements [1]. Around 1 % of these are reported to be complicated by post-operative infection [2]. In addition, there are around 5,000 diabetic foot infections with associated osteomyelitis and a smaller number of infections of the axial skeleton. Treatment costs for bone and joint infections are estimated to be between £20,000 and £40,000 per patient [3–5].

Many consider a prolonged course (4–6 weeks) of intravenous (IV) antibiotics the “gold standard” treatment for bone and joint infections [6–8]. However, such practice derives from an era, prior to properly embedded pharmacokinetic principles, during which a widely held misconception that IV therapy is “stronger than oral therapy” was established. As a result, IV antibiotic therapy is often preferred to oral therapy and has become an accepted standard of care even for many non-acute infections. There is very limited evidence to support this practice. On the contrary, there is a growing body of literature, from both pharmacokinetic studies and from clinical outcome studies, to suggest that a wide range of infectious diseases (for example, pneumonia [9], urinary tract infections [10], low-risk neutropenic sepsis [11], skin and soft tissue infections [12] and endocarditis caused by *Staphylococcus aureus* [13]) can be effectively treated with oral therapy in the post-acute setting. Provided that agents are carefully chosen with respect to bioavailability and tissue penetration, there is no biologically plausible reason to believe that bone and joint infections should be any different.

A Cochrane review of five small trials involving a total of 180 participants with bone or joint infection showed no benefit of IV as compared to PO therapy [14], although the authors conclude that there is currently insufficient evidence to inform a widespread change in practice. Larger observational studies have reported high success rates for prosthetic joint infection with a shortened course of IV antibiotics or use of antibiotic cement spacers [15, 16], but observational comparisons are prone to confounding by indication.

Prolonged IV antibiotic therapy mandates placement of an intravenous vascular access device, which carries a risk of complications such as catheter-related infection and thromboembolic disease. Oral antibiotic therapy mitigates such risks [17, 18], is more convenient for the patient and is less costly. On the other hand, oral therapy carries a greater risk of poor adherence, gastrointestinal intolerance and variable serum levels related to drug bioavailability. Although clinicians can usually identify an appropriate oral antibiotic regimen with high oral bioavailability and good tissue penetration, this strategy has not yet been compared to intravenous treatment in a large clinical trial.

We conducted a single centre pilot study that concluded in March 2013 (Randomised open label study of oral versus intravenous antibiotic treatment for bone and joint infections requiring prolonged antibiotic treatment: Preliminary study in a single centre, NCT 00974493, Eudract Number: 2009-015744-42). The results were reviewed by an independent data monitoring committee (DMC), which advised that it was safe and appropriate to extend the trial. Thereafter, the trial switched to multicentre recruitment, and we transferred the data from the 228 participants in the pilot study to the multicentre trial database.

## Methods

### Study hypothesis and objectives

We hypothesise that per oral (PO) antibiotic therapy is non-inferior to IV antibiotic therapy in the treatment of bone and joint infection, as judged by the proportion of patients experiencing definite treatment failure during one year of follow-up. Our secondary objectives include: assessment of safety and tolerability (including serious adverse events, all-cause mortality, line complications, *Clostridium difficile* infection, early termination of allocated strategy); adherence to the PO antibiotics; a health economic analysis; patient reported outcome measures (PROMs) (Oxford Hip Score [OHS] and Oxford Knee Score [OKS] when appropriate, and EQ-5D-3 L); and possible or probable treatment failure as composites with definitive treatment failure.

The trial is in full compliance with the Helsinki Declaration and has ethical approval (REC reference 09/H0604/109 for the single centre preliminary study and REC reference 13/SC/0016 for the multicentre trial) from the NHS Health Research Authority.

### Trial participants

Participants are recruited from 30 secondary care centres, all of which are NHS hospitals in England or Scotland. All sites currently use 6 weeks of IV antibiotic therapy as their standard initial treatment for some or all categories of bone and joint infection, and all are able to deliver IV antibiotics to patients after discharge from hospital.

Participants are considered for inclusion when an infection specialist reviews a patient with a bone or joint infection. The contact is triggered by routine care pathway, for example, following referral by a surgical team, a referral from primary care direct to infectious disease services, or by following up a laboratory result. Informed consent is obtained from each participant by good clinical practice (GCP) trained research staff after assessing their understanding of the Patient Information Sheet (PIS) [see Additional files 1 and 2]. Eligibility is determined by the inclusion and exclusion criteria listed below.

### Inclusion criteria

The participant may be enrolled only if he or she meets *each* of the following criteria:

1. Has a clinical syndrome comprising any of the following: a) localised pain or b) localised erythema or c) temperature >38.0 °C or d) a discharging sinus or wound
2. Is willing and able to give informed consent
3. Is aged 18 years or above
4. Has received 7 days or less of intravenous therapy after an appropriate surgical intervention to treat bone or joint infection (regardless of pre-surgical antibiotics) or, if no surgical intervention is required, the patient has received 7 days or less of intravenous therapy after the start of treatment for the relevant clinical episode
5. Has a life expectancy > 1 year
6. Has a bone and joint infection in one of the following categories: a) native osteomyelitis (bone infection without metalwork) including haematogenous or contiguous osteomyelitis; b) native joint sepsis treated by excision arthroplasty; c) prosthetic joint infection treated by debridement and retention, by one stage revision or by excision of the prosthetic joint (with or without planned re-implantation); d) orthopaedic device or bone-graft infection treated by debridement and retention or by debridement and removal; e) spinal infection, including discitis, osteomyelitis or epidural abscess.

### Exclusion criteria

The participant may not enter the study if he or she has *any* one of the following:

1. *Staphylococcus aureus* bacteraemia on presentation or within the last 1 month
2. Bacterial endocarditis, either on presentation or within the last month (NB: there are no study mandated investigations, so participants are not required to have echocardiograms, blood cultures, or any other investigations to exclude endocarditis in the absence of a clinical indication)
3. Any other concomitant infection which, in the opinion of the clinician responsible for the patient, requires a prolonged course of intravenous antibiotic therapy (for example, mediastinal infection or central nervous system infection)
4. Mild osteomyelitis, defined as bone infection which, in the opinion of the clinical investigator, would not usually require a 6-week course of intravenous antibiotic therapy
5. An infection for which there are no suitable antibiotic choices to permit randomisation between the two arms of the trial (for example, where organisms are only sensitive to intravenous antibiotics)
6. Previous enrolment in the trial
7. Septic shock or systemic features requiring intravenous antibiotic therapy in the opinion of the treating clinician (the patient may be re-evaluated if these features resolve)
8. Evidence of being unlikely to comply with trial requirements following randomisation in the opinion of the investigator
9. Clinical, histological or microbiological evidence of mycobacterial, fungal, parasitic or viral aetiology of the infection
10. Receiving an investigational medical product as part of another clinical trial

The use of antibiotic-loaded cement in spacers, bone substitutes or beads at the site of infection is not an exclusion criterion, but is recorded at baseline. Pregnancy, renal failure and liver failure are not exclusion criteria, provided suitable antibiotic options can be identified for both IV and PO therapy prior to randomisation.

### Randomisation

An electronic randomisation service, with telephone backup if necessary, is provided through a clinical trials unit. After confirming the patient’s eligibility, the randomisation service assigns a sequentially allocated study number and informs the investigator of the treatment allocation in real time and by confirmatory e-mail. Randomisation is stratified by site, to take account of variation in clinical practice among centres.

The local clinician or study nurse is responsible for documenting the participant’s enrolment in their clinical notes and for informing the participant’s general practitioner.

### Minimising bias

Blinding is not possible, since we consider that giving prolonged intravenous placebo would pose an unnecessary risk to participants and therefore would be unethical. Because an open label study is at risk of bias, we have appointed an endpoint review committee to review the clinical notes relating to any patient who fulfils the criteria for a potential treatment failure. All notes reviewed are redacted for personal identifiable data and for any information that might indicate the choice and route of antibiotic therapy. The endpoint review committee is therefore blind to treatment allocation. The notes are examined against objective criteria for meeting the primary endpoint.

### Study interventions: PO versus IV antibiotic strategy

The selection of individual antibiotic agents within the allocated strategy (that is, PO or IV antibiotics) is the responsibility of the infection specialist caring for the patient; the choice depends on microbiological assessment, the side effect profile and patient preference. In the event of a culture-negative bone or joint infection (or where there is a delay in availability of culture results), the infection specialist selects the most appropriate empiric therapy. If new information becomes available, the infection specialist may change the choice of antibiotic agent but should remain within the allocated route of administration strategy; if this is not possible, the participant will have reached an endpoint.

If randomised to IV strategy, participants are expected to complete 6 weeks of IV antibiotic therapy. Where it is common practice to use adjunctive oral agents in patients treated with IV therapy (for example, oral rifampicin as adjunctive therapy for biofilm-related staphylococcal infection), such therapy will be allowed.

If randomised to the PO strategy, participants are expected to commence PO therapy within seven days of definitive surgical management (or, if managed without surgery, from the start of antibiotic therapy for that clinical episode). If a participant allocated to the PO strategy requires IV antibiotic therapy for an unrelated intercurrent illness during the initial 6 weeks of treatment, or experiences vomiting or inability to swallow, or concerns arise about absorption of oral medication, then IV antibiotic therapy may be substituted for up to five days. If IV antibiotic prescribing exceeds five days, the patient will have met a secondary endpoint, but will still contribute to the intention-to-treat analysis.

Follow-on antibiotic treatment after the initial 6 weeks may be prescribed in either arm of the trial, but the choice of agent, duration and route of administration are not governed by the trial protocol.

If continuing in the randomised strategy (IV or PO) is no longer compatible with optimal clinical care, the clinician must commence appropriate alternative therapy. Appropriate reasons for discontinuing the allocated treatment might include lack of suitable medication within the allocated strategy, adverse reactions, contraindications or susceptibility testing results. Failure to maintain intravenous access is an appropriate reason for discontinuing IV antibiotics. A wound discharge or other clinical signs related to the incident infection, or its apparent resolution, are not appropriate indications for changing from one treatment strategy to the other, since there is equipoise regarding efficacy.

There are no formal withdrawal criteria in this study other than at the request of a participant. All patients are free to withdraw their consent at any time; if they elect to withdraw from the allocated treatment strategy during the randomised treatment phase, they will meet a secondary endpoint but may still be followed up and be included in the analysis provided that appropriate consent is given.

Any medical decision to withdraw a participant from the randomised strategy is discussed with the chief investigator (CI) or the trial physician. Changing the antibiotic whilst remaining within the allocated strategy need not be discussed, but a clinician with appropriate training in managing infection must make such a decision.

**Fig. 1:**
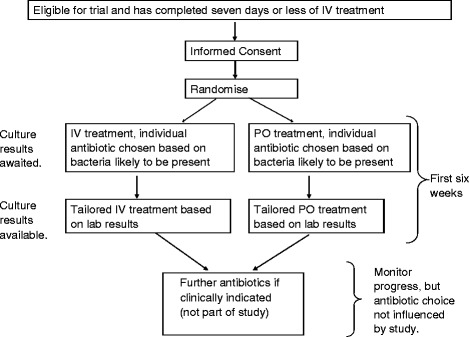
Participant pathway for the OVIVA trial

The infection specialist may adjust dosage based on renal or hepatic function, drug interactions or other factors according to drug labelling information, the British National Formulary and local pharmacy guidelines. All antibiotics used (including dosage, route of administration and duration) are recorded from the day of randomisation through to final follow-up at one year (Fig. 1).

### Assessments

When a participant is an inpatient, the study clinician or research nurses will maintain contact with the clinical team to identify potential endpoints, and to ensure implementation of the randomised antibiotic strategy. Following hospital discharge, participants are seen according to clinically determined follow-up plans. Trial-specific data are obtained from the case record at 6 weeks (range from day 21 to day 63), 4 months (range day 70 to day 180) and 1 year (range day 250 to day 420). If the patient does not attend clinic within the specified date ranges, the investigator arranges a telephone review with the participant or the participant’s general practitioner (GP) to identify potential endpoints or serious adverse events. If, based on the telephone discussion, clinical review is indicated, the investigator organises this and advises the patient accordingly.

A study clinician reviews the source documents from routine care visits when completing investigator reviews. They record:

- Microbiology and histology results and date of discharge (first review only)
- Outpatient visits since randomisation
- Serious adverse events
- Re-admissions for inpatient care
- Type of intravenous catheter (line) used and any line-related complications
- Episodes of *Clostridium difficile* associated diarrhoea
- Antibiotic use to date (including dosage, route and model of care, for example, district nurse, self-administered or daily clinic visits)
- Presence or absence of any potential endpoints
- Reasons for not completing the planned antibiotic course (if applicable).

### Outcome measures

Endpoints are identified by prospective surveillance throughout the year post-randomisation.

The primary endpoint is definite failure of infection treatment, where definite failure is indicated by one or more of the following:

- Isolating bacteria from two or more samples of bone/spine/peri-prosthetic tissue, where the bacteria are phenotypically indistinguishable
- Isolating a pathogenic organism (for example, *Staphylococcus aureus,* but not *Staphylococcus epidermidis*) on a single, closed biopsy of native bone or spine
- Diagnostic histologic findings on bone or peri-prosthetic tissue
- Formation of a draining sinus tract arising from bone or prosthesis
- Recurrence of frank pus adjacent to bone or prosthesis.

Secondary endpoints are:

1. Serious adverse events (SAEs), including death (that is, all-cause mortality)
2. IV line complications (infection, thrombosis or other events requiring early removal or replacement of the line)
3. “Probable” or “possible” treatment failure, as composites with definite treatment failure. These are determined by blinded endpoint committee review and defined by the presence of any one of the following criteria:

- Loosening of a prosthesis, confirmed radiologically
- Non-union of a fracture after 6 months, confirmed radiologically
- Superficial spreading erythema, treated as cellulitis with an antibiotic for >1 week; where results from deep tissue samples do not meet the primary endpoint as described above

Where appropriate deep tissue samples have been sent for microbiology and results of the culture are negative, and any of a), b) or c) are met, then the endpoint will be regarded as “possible”. On the other hand, where deep tissue samples are not sent for microbiology, and any of a), b) or c) are met, then the endpoint will be regarded as “probable”

4. Early termination of the planned 6-week period of oral or IV antibiotic therapy because of adverse events, patient preference or any other reason
5. Resource allocation determined by: a) length of inpatient hospital stay; b) frequency of outpatient visits; or c) antibiotic prescribing costs
6. Quality of life evaluated by EQ-5D-3 L questionnaire
7. Oxford Hip and Knee Scores (where infection is in the hip or knee)
8. Adherence to oral medication.

The study clinicians determine secondary endpoints 1, 2, 4 and 5. The blinded endpoint review committee determines primary endpoints and secondary endpoint 3, by using redacted notes. Participant questionnaires determine secondary endpoints 6 and 7. Secondary endpoint 8 is determined by questionnaire in all centres, and by medication event monitoring systems (MEMS) at four sentinel sites.

### Endpoint review committee

The endpoint review committee (ERC) is composed of three independent clinicians (two infectious disease specialists and one orthopaedic surgeon) with expertise in the management of orthopaedic infections.

The hospital notes relating to the potential endpoint are redacted for both personal identifiable information and specifics of antibiotic treatment or IV line insertion, which could indicate the route of administration of antibiotics. The redacted notes are forwarded to the ERC, who determine whether an endpoint has been met, either by consensus or by a vote called by the chair if consensus cannot be reached.

The ERC is only required to review definite or potential treatment failures. All other endpoints are determined directly by the local study clinicians.

### Adherence and medication event monitoring systems (MEMS)

Patient adherence to antibiotic therapy may directly influence the outcome of treatment. All participants randomised to the oral strategy therefore receive questionnaires to monitor adherence. A subgroup of participants randomised to the intravenous strategy and taught to self-administer also receive adherence questionnaires. In order to avoid intrusion and to minimise undue influence on patient behaviour, participants do not receive any direct antibiotic adherence support (such as text message reminders or telephone monitoring), but the importance of adherence is explained at the time of recruitment and reinforced at the time of discharge. The Patient Information Sheet (PIS) includes information written by the patient representatives which explains the importance of, and rationale behind, medication adherence.

In order to validate the adherence questionnaire returns, selected sites (Oxford University Hospitals, Guy’s and St. Thomas’ Hospitals, The Royal National Orthopaedic Hospital and The Royal Free Hospital, London) dispense oral antibiotics in pill containers with MEMS. This method of monitoring has become standard in studies of medication where adherence is critical [19, 20]. Sensors in the caps detect opening and closing, and record these events with a date stamp. The sensors are read at a later date to verify whether patients opened and closed their bottles at times that are consistent with their prescription. MEMS are used only with specific consent from participants. If more than one antibiotic is prescribed, we use the MEMS sensors on the more frequently dosed antibiotic. If changes to antibiotic prescriptions are required after discharge, trial participants continue with only paper adherence questionnaires.

### Safety

Since the OVIVA trial does not involve randomisation to a specific therapy, it is not a “Clinical Trial of an Investigational Medicinal Product,” as defined by the European Union (EU) directive 2001/20/EC. Safety reporting therefore refers to the trial sponsor and the DMC. We record all SAEs identified within a year of randomisation. We consider episodes of definite or potential treatment failure as SAEs.

If an investigator becomes aware of an unexpected SAE during the trial, he or she contacts the CI, who clarifies clinical details and reports the SAE to the sponsor. If, in the opinion of the CI or the sponsor, an unexpected SAE may be relevant to participant safety, a detailed report including an assessment of causality and severity is forwarded to the DMC, which in turn makes a recommendation regarding the safety of the trial in the light of this report.

Expected SAEs that do not undergo expedited reporting are defined as:

- Complications of bone/joint surgery
- Complications of the bone/joint infection for which the patient is undergoing treatment (including potential endpoints)
- Drug reactions as detailed in the product literature (that is, the summary of product characteristics (SMPC) or British National Formulary)
- Drug reactions for concurrent medications given for routine clinical care as detailed in the product literature (the SMPC or British National Formulary)
- Intercurrent illness causally related to the comorbid conditions that the investigator believes are likely diagnoses, given the patient’s history, age and other factors.

The investigators use their judgement, such that SAEs technically meeting definitions above for expectedness, but that seem unexpected in terms of severity, duration or other factors, may be reported as unexpected.

### Sample size

The sample size estimation of 1,050 was based on an anticipated overall failure rate of 5 %, as suggested by short-term follow-up in the single centre pilot study, and a non-inferiority margin of 5 % (a relative increase of 100 %), with a one-sided alpha = 0.05, 90 % power and 10 % loss to follow-up.

Pooled data from a planned interim analysis demonstrated that the true event rate is likely to be closer to 12.5 %. To account for this, we adjusted the non-inferiority margin to 7.5 % (a relative increase of 60 %). As the final control group failure rate remains unknown, recruitment will continue as planned until October 2015 to achieve the largest possible sample size within the original target, and to optimise the potential utility of subgroup analyses. The DMC and ethics committee approved this amendment.

Sample size calculations for binary outcomes were performed in Stata (StataCorp LP, College Station, TX, USA).

### Analysis of efficacy

#### Primary endpoint

Based on intention to treat, the proportions of participants experiencing the primary endpoint at one year follow-up (definitive treatment failure as adjudicated by a blinded ERC) will be tabulated by treatment administration group (that is, PO versus IV therapy). If the absolute, upper 90 % confidence interval around the unadjusted difference (oral versus intravenous) is less than 7.5 %, then the criteria of non-inferiority will be met.

#### Secondary endpoints

Secondary analyses will include: (i) a per-protocol analysis based on all participants who have received at least 4 weeks of randomised therapy and (ii) intention-to-treat and per-protocol analyses in the subgroup with “definite” or “definite”/“probable” infection at randomisation. These secondary analyses will focus on consistency of point estimates and 95 % CI, rather than formal comparison with the 7.5 % non-inferiority margin. We will similarly compare the proportions of participants with secondary endpoints, or the distributions of continuous secondary outcomes (rank sum tests). Subgroup analyses will use interaction tests to determine the consistency of treatment effects by type of infection and infecting pathogen. We record treatment intentions for both intravenous and oral routes at baseline before randomisation. Subgroup analysis will compare efficacy of intravenous versus oral antibiotic therapy according to whether or not rifampicin was used as an adjunctive therapy in the intravenous and oral arms (four subgroups). We will also conduct subgroup analyses according to the clinician’s specific antibiotic intentions, as recorded prior to randomisation.

A survival analysis will be performed to assess post-randomisation surveillance bias, which would present as a delay in time to meeting an endpoint in one randomised group. Other secondary analyses will include regression models (logistic (binary) or quantile (continuous)) to calculate estimates of treatment differences for the primary and secondary endpoints adjusted for age, comorbidity, infecting pathogen and type of surgical intervention.

The statistical analysis plan will be locked prior to analyses being undertaken and will be made freely available at the time of publication.

#### Patient adherence

We will describe patient adherence according to the Morisky scale, using data from the questionnaires (full cohort) and MEMS (sentinel sites).

#### Diagnostic subgroup definitions

The clinical diagnostic inclusion criterion means the trial reflects real-world practice, and facilitates timely entry to the study. In the analyses we will use histology, microbiology and clinical details to determine “definite” evidence of infection, as defined by: a) isolating bacteria from two or more samples of bone/spine/peri-prosthetic tissue, where the bacteria are phenotypically indistinguishable OR b) a pathogenic organism (for example, *Staphylococcus aureus* but not *Staphylococcus epidermidis*) on a single, closed biopsy of native bone or spine OR c) diagnostic histology on bone/peri-prosthetic tissue OR d) a draining sinus tract arising from bone/prosthesis or OR e) frank pus adjacent to bone/prosthesis.

If any of these criteria are met, then the category “definite” infection is applied without independent review.

Where these criteria are not met, the review committee members are sent a redacted copy of the patient’s admission notes and laboratory results from the time of randomisation, and apply the following criteria to determine “probable” or “possible” infection.

Infection is categorised as “probable” where microbiological sampling has not been undertaken, plus none of the other criteria for definite infection are fulfilled and any one of the following are met:

- Radiological or operative findings of periosteal changes suggesting chronic osteomyelitis
- Radiological findings suggesting discitis/spinal infection
- Development of a discharging wound after an orthopaedic procedure where prosthetic material has been implanted
- Presence of deep pus close to but not adjacent to bone/prosthetic joint/orthopaedic device
- Presence of peri-prosthetic necrotic bone
- Rapid loosening of a joint prosthesis/orthopaedic device (that is, leading to localised pain in less than 3 months since implantation) in the absence of a mechanical explanation for rapid loosening.

Infection is categorised as “possible” where microbiological sampling has been undertaken with negative results (according to criteria described above for “definite” infection) plus other criteria for definite infection are not fulfilled and, in addition, one or more of the criteria listed a) to f) above is met. The review committee members are blinded to treatment allocation and subsequent outcome. Secondary analysis will evaluate non-inferiority for “definite” or “definite”/“probable” infections only.

### Health economic analysis

The health economic evaluation has two parts. The first, a within-trial analysis, will be performed based on the resource use and health-related quality of life (EQ-5D-3 L) data. We will use the British National Formulary for antibiotic costs (with a sensitivity analysis for hospital pharmacy discounts). We will include the costs associated with IV administration based on staffing requirements, equipment cost, clinic visits and transport costs for patient visits as observed in the trial. For unplanned inpatient stays and additional outpatient attendances other than those related to IV administration, we will use standard NHS reference costs.

We will calculate mean costs in each arm of the trial and differences in costs between the two arms, with 95 % confidence intervals. The EQ-5D-3 L instrument will be used to estimate per-patient quality-adjusted life years (QALY) with adjustment for any differences between the groups in EQ-5D-3 L at baseline. Non-parametric bootstrapping techniques will be employed to confirm the robustness of the statistical analysis of cost, QALY and cost per QALY. Uncertainty in cost-effectiveness will be represented on the cost-effectiveness plane and as confidence intervals for cost-effectiveness ratios, or as cost-effectiveness acceptability curves, as appropriate.

The second part of the analysis will be to extrapolate the observed results in OVIVA beyond the clinical trial, in order to explore the potential lifetime cost-effectiveness of a switch in antibiotic administration route strategy. This extrapolation will be made in each diagnostic group, using estimates of long-term recurrence from the literature and the observed recurrence rates observed within the period of the trial. We will also use the published longer term costs associated with disability in order to reflect the consequences of treatment failure that persist beyond the end of the trial. Taking these estimates together, we will extrapolate the costs beyond the period of observation within the year of follow-up in the trial. This will necessarily involve a series of assumptions in applying estimates from the literature, and extensive sensitivity analyses will be examined in order to explore the robustness of the estimates.

### Trial management and quality assurance procedures

The study is conducted in accordance with the current approved protocol, International Conference on Harmonisation (ICH) Good Clinical Practice guideline, relevant regulations and standard operating procedures, including data protection.

We undertake remote monitoring of data entered in real time on a secure, anonymised database and conduct monitoring visits of collaborator sites to confirm integrity of data.

### Data monitoring committee

The DMC is composed of three members, two of whom are specialists in infectious diseases and the other a senior statistician. None are involved in recruitment, randomisation or follow-up for trial participants or contribute to the trial in any way other than through the DMC. The DMC met to discuss the study design and standard operating procedures shortly before the start of the study. The DMC evaluate the frequency of endpoints in an unblinded analysis annually in a closed meeting and make recommendations to the trial steering committee. Although the DMC may recommend suspension or cessation of the trial at any time, it is expected that they would only recommend early stopping if there was a very significantly worse outcome in the PO antibiotic group as compared to the IV group, as determined for example by the Haybittle-Peto stopping boundary.

### Trial steering committee

The trial steering committee (TSC) consists of two independent co-chairs (Graham Cook, Imperial College London and John Paul, Health Protection England), two public/patient group representatives (Fraser Old, Nuffield Orthopaedic Centre Network and Jennifer Bostock, Healthcare-Associated Infection Service Users Research Forum), and the chief investigator, supported by the trial physician, coordinator and statistician.

The TSC met first at the start of the trial, and then meets yearly to review recruitment rates, protocol amendments, and any protocol deviations identified. The co-chairs of the TSC receive recommendations directly from the DMC and may make recommendations to the sponsor regarding the running of the trial.

### Ethical considerations

All clinicians involved in the study have acknowledged a position of equipoise in relation to route of antibiotic therapy for treatment for bone and joint infections; they accept that there is currently insufficient evidence to determine whether oral antibiotics are inferior (or superior) to intravenous antibiotics in this context. This uncertainty is conveyed to patients both verbally and in writing. All investigators have agreed that they will ensure that this study is conducted in full conformity with relevant regulations and with the ICH Guidelines for Good Clinical Practice (CPMP/ICH/135/95) July 1996. The trial is in full compliance with the Helsinki Declaration and received a favourable ethical opinion from the Health Research Authority through the NRES Committee South Central - Oxford B (REC reference 13/SC/0016).

### Funding

The trial is supported by grant funding from the National Institute for Health Research (NIHR) Health Technology Assessment (HTA) Programme (11/36/29).

## Discussion

Despite a long-standing view that the successful management of bone and joint infection depends upon the use of intravenous antibiotics [21–23], there is no evidence to suggest that oral antibiotic therapy results in worse outcomes. Indeed, a Cochrane review of five comparative trials (180 patients in total) of antibiotic therapy for chronic osteomyelitis demonstrated no benefit of IV versus oral antibiotic therapy, although the authors concluded that there was insufficient evidence to inform clinical practice [14]. The Infectious Diseases Society of America guideline on the management of prosthetic joint infections [24] and a review by Fraimow [25] suggest the use of highly bioavailable oral agents may be an appropriate alternative to intravenous therapy, provided patient factors do not limit the drug’s pharmacokinetic properties. Despite these recommendations, there is significant variation in practice, with some centres advocating prolonged courses of IV therapy, some using short courses of IV therapy, and others relying primarily on locally administered antibiotic agents [26, 27]. This lack of consensus demonstrates that the current trial addresses an important hypothesis and that the results are likely to influence practice. We therefore set out to define whether or not oral therapy is non-inferior to IV therapy in the management of bone and joint infection.

The OVIVA trial is pragmatic in that it is fully embedded into usual care and, as far as possible, reflects standard practice in all respects other than randomisation of treatment strategy and data collection. No additional diagnostic investigations, trial-specific clinic visits or blood tests are required of the participants. This has the advantage of reducing the influence of possible differential observer effects by treatment arm.

Intravenous therapy has several potential disadvantages as compared to oral therapy. Use of IV therapy may lead to a delay in discharge from hospital, either whilst awaiting insertion of an intravenous access device and setting up outpatient parenteral antibiotic treatment (OPAT) or, in the absence of an OPAT service, hospitalisation for the entire duration of therapy. In addition, unless they learn to self-administer, many patients find it inconvenient to either attend the hospital daily or to arrange access in their own homes to a visiting nurse. Complications related specifically to IV therapy include line fracture or blockage, bleeding, thromboembolic events and line-related infections. The latter carries with it a crude mortality estimate of up to 25 % [28]. Intravenous therapy is more costly than oral therapy; we estimate the average cost of 6 weeks of IV therapy administered through an OPAT service in the UK to be 10 times that of an equivalent oral therapy (£2,000 versus £200). We aim to address each of these factors through patient questionnaires (PROMs data), comparisons of length of stay and a full health economic analysis.

Oral therapy also carries some disadvantages. Agents need to be chosen carefully, taking into account bioavailability and achievable tissue levels. Gastrointestinal adverse effects are likely to be more common with oral therapy, and patients are likely to have less nursing or medical supervision as compared to patients on IV therapy. More importantly, adherence to therapy is plausibly better with supervised IV therapy than with self-administered oral therapy; this is a major concern when treating an infection for which tissue concentration of antibiotics is critical. In order to address this, patients in the OVIVA trial are provided with written information explaining the importance of adherence and are asked to complete adherence questionnaires during the first 6 weeks of therapy. To validate the questionnaires, under additional consent, oral medication is dispensed in MEMS bottles at four sentinel recruitment sites.

There is reasonable evidence supporting the effectiveness of oral therapy in several other infectious processes that are traditionally treated with IV therapy. These include selected cases of bacterial endocarditis [13], MRSA skin and soft tissue infections [12] and febrile neutropenia [29]. Pathophysiologically, there is no reason to believe that results of treatment of bone or joint infections should be different, provided that appropriate oral agents are selected.

If oral therapy is shown to be non-inferior to intravenous therapy for bone and joint infection, the findings of this trial are likely to benefit patients, the NHS and the health economy.

A further potential benefit of targeted oral therapy includes a reduction in use of broad-spectrum antibiotics and the consequent risk for emerging antibiotic resistance.

Finally, there is a clear mandate from the Department of Health to ensure patient-centred treatment including, where possible, limitation of hospital attendances, promotion of an independent ”normal life style” and greater patient choice over their own treatment [30].

There are some limitations associated with the trial.

The OVIVA trial is an open label study. The decision to use this design was based on two principles. Firstly, exposure of patients to a placebo IV therapy for a period of up to 6 weeks would pose unnecessary risks associated with the use of an intravascular access device and would therefore be unethical. Secondly, due to the number of different antibiotics required to provide optimal care for all patients randomised, it was not feasible to provide matched placebos in every case. Although an open label design leaves the trial open to bias, almost certainly in favour of IV therapy, the endpoints are determined according to predefined criteria by an independent committee who are blinded to treatment allocation. This is achieved through redaction from case notes of any information that might betray the treatment allocation (for example, reference to IV access devices, OPAT, drug names, therapeutic drug monitoring). Primary endpoints are defined by objective clinical and microbiological criteria, assessment of which requires attendance at, or admission to, hospital. They are therefore hard endpoints, the interpretation of which is unlikely to be influenced by treatment allocation or other confounding factors.

OVIVA is an inclusive study; there is no selection by organism, procedure or surgical site. We recognise that this results in a heterogeneous study population, but believe that the advantages of generalisability outweigh the disadvantages. A more selective recruitment strategy, such as inclusion only of primary arthroplasty infections, would have eliminated concern around heterogeneity. However, with an eligible annual population of around 1,500 in the UK, recruitment to such a trial would have been prohibitively long, and the question would remain unanswered for many of the circumstances under which we manage bone and joint infection in real life. Secondly, the hypothesis of the trial is based strictly upon the pharmacokinetic principle that appropriately selected oral antibiotics can provide similar tissue concentrations as compared to intravenous antibiotics. This principle is highly unlikely to be influenced by a differential effect determined by, for example, the site of infection, the presence or absence of metalwork, the number of previous infections or the extent of surgical debridement. Thirdly, the randomisation process should ensure that heterogeneity will be matched across the two arms. Where possible, subgroup analyses will aim to identify any significant differential effect within defined populations. Because there are numerous recruiting centres, ranging from specialist units to district general hospitals, the data collected should be an accurate representation of patients and practice across the NHS.

Eligibility for recruitment to the trial is based upon clinical criteria rather than diagnostic laboratory results. There are several reasons why we have not included histological or microbiological results as part of the inclusion criteria. Firstly, between 12–28 % of bone and joint infections diagnosed clinically are not confirmed in the laboratory [31, 32], as a result, for example, of prior exposure to antibiotics or sampling error. Nonetheless, these patients are treated as infection based on clinical criteria. Secondly, the results of laboratory tests, particularly the histology results, are not always complete within seven days of sampling; had we relied upon laboratory results as part of the inclusion criteria, many patients would have had to be excluded from this trial on account of this delay. Thirdly, the pragmatic design of this trial gives due authority over clinical management to the surgeon or physician responsible for the patient. If, according to a research definition, infection was deemed not present, the trial could potentially undermine a clinician’s autonomy to treat an infection based on clinical criteria alone. Finally, in order to account for the possibility that uninfected patients are included, every case which fails to meet a strict prospective definition of infection is reviewed by an independent committee for a consensus decision on their infection status at the time of recruitment. The results from these reviews will be reflected in the presentation of results.

There are two circumstances in which an apparent deviation from allocated treatment arm might arise. Firstly, for participants randomised to IV therapy, the use of purely adjunctive oral agents such as rifampicin is allowable. This may at first seem counterintuitive in a study which aims to compare IV with oral therapy but is based upon common practice outside the context of the trial. The principle stems from the fact that, for some oral agents, bioavailability is close to 100 %. There is therefore no scientific rationale to suggest any advantage of IV therapy for these agents. Examples include oral rifampicin, which is routinely used alongside IV therapy in the management of biofilm-associated staphylococcal disease, and metronidazole, which is commonly used in polymicrobial osteomyelitis. To exclude patients allocated to the IV arm but who require adjunctive oral therapy would likely incur a bias in favour of oral therapy. Secondly, participants randomised to the oral arm are allowed up to five days of IV therapy to allow for treatment of intercurrent illness or for short periods where, for unrelated reasons, oral therapy is not appropriate. It was not designed to be used as a rescue treatment for the bone infection under therapy; the protocol makes this very clear. To withdraw patients on the grounds that they had an unrelated concomitant illness which, in the opinion of a physician independent of the trial, required IV therapy might be considered discriminatory or unethical by some readers. Given that all patients will have been prescribed at least 6 weeks of therapy for the incident bone infection, we think that a short course of IV therapy in a small minority of patients is unlikely to significantly influence the results. To ensure transparency around both of these circumstances, all antibiotic use (including dose, route of administration and duration) will be recorded from the day of randomisation through to one year follow-up, and appropriate analyses will be presented.

Initial recruitment of study patients was slower than anticipated. This was primarily due to delays in site setup and in establishing mechanisms for the timely identification and referral of potential participants to the research teams. Recruitment was also adversely affected by a surprising number of patients who, having read the PIS and understanding the principle of equipoise, requested oral therapy rather than IV therapy. In order to achieve the recruitment target, we implemented a web-based network site, monthly teleconferences and recruitment drives, and requested an extension without additional cost to the funding agency.

At the second planned interim analysis, the overall primary endpoint event rate (12.5 %) was significantly higher than had been predicted from the single centre pilot study (5 %); reasons for this might include stochastic variation related to a small sample, the effect of recruitment at a single specialist centre or a combination of factors. In order to accommodate this unexpected finding, and with appropriate ethical permission and endorsement from the DMC, we elected to amend the non-inferiority margin from 5 % to 7.5 %. This figure remains within published guidelines, which suggest that a non-inferiority margin of 10 % is appropriate for most therapeutic intervention trials in infection [33]. We therefore believe that the non-inferiority margin remains appropriate for this trial and is unlikely to jeopardise the utility of the results.

Despite its limitations, the trial will be the largest study of its type addressing this question of oral versus IV antibiotic therapy in bone and joint infection, and is likely to have important implications for patients and healthcare practitioners in the field of orthopaedic infection, and for the health economy.

## Trial status

The OVIVA trial started in March 2013. Initial site setup and recruitment were slower than anticipated, and therefore a one-year extension was recommended by the TSC and DMC and agreed to by the funder. Recruitment will cease in November 2015, and the trial will close in March 2017.

## Additional files

Additional file 1:
**OVIVA Patient Information Sheet.** Information sheet provided to patients prior to enrolling in the trial. (PDF 127 kb) Additional file 2:
**OVIVA Consent Form.** Form used in the OVIVA trial to record informed consent. (PDF 13 kb)

## Abbreviations

CI: chief investigator
CRF: case report form
DMC: data monitoring committee
ERC: endpoint review committee
GCP: good clinical practice
GP: general practitioner
HTA: Health Technology Assessment
ICH: International Conference on Harmonisation
IV: intravenous
MEMS: medication events monitoring system
NHS: National Health Service
NIHR: National Institute for Health Research
OHS: Oxford Hip Score
OKS: Oxford Knee Score
OPAT: outpatient parenteral antimicrobial therapy
PIS: Patient Information Sheet
PO: per oral
PROM: patient reported outcome measure
QALY: quality-adjusted life years
REC: Research Ethics Committee
SAE: serious adverse event
SMPC: summary of product characteristics
TSC: trial steering committee
