# Supplementary material for: Oral versus intravenous antibiotic treatment for bone and joint infections (OVIVA): study protocol for a randomised controlled trial
Source: Trials. 2015 Dec 21;16:583. doi: 10.1186/s13063-015-1098-y (PMC4687165; doi:10.1186/s13063-015-1098-y)
Supplement: Additional file 1: — OVIVA Patient Information Sheet. Information sheet provided to patients prior to enrolling in the trial. (PDF 127 kb) [file 13063_2015_1098_MOESM1_ESM.pdf]

PATIENT INFORMATION SHEET

Oral versus intravenous antibiotics for bone and joint infections

(Full Title: Randomized open label study of oral versus intravenous antibiotic treatment for bone and joint infections requiring prolonged antibiotic treatment: multi-centre study.)

Acronym: OvIVA (Oral versus Intra Venous Antibiotics)

We would like to invite you to take part in our research study. Before you decide we would like you to understand why the research is being done and what it would involve for you. One of our team will go through the information sheet with you and answer any questions you have. This should take about 30 minutes, but please take longer if you need to and feel free talk to others about the study if you wish.

This study is about comparing two different methods of giving antibiotics to patients with bone and joint infection. Antibiotics will either be given intravenously (injected into a vein) or given orally (as tablets).

Part 1 of this information sheet gives general information about the study and what is involved for patients who choose to take part. Part 2 provides more detailed information regarding confidentiality, study results, and what to do if things go wrong.

Please ask us if anything is unclear.

Part 1: General Information about the OvIVA study

What is the purpose of the study?

In many hospitals doctors use intravenous antibiotics for 6 weeks to treat bone and joint infections; in other hospitals, doctors prescribe mainly tablets. Each route has its advantages and disadvantages but we do not yet know whether one is better than the other.

We are therefore going to test two strategies for using antibiotics; a) intravenous antibiotics for 6 weeks or b) oral antibiotics to complete 6 weeks after only a short period (less than 7 days) on intravenous therapy. All of the antibiotics used in this trial are currently licensed for use in the UK, and no experimental drugs will be used.

In some situations, the doctors looking after you may ask you to continue to take antibiotics after the initial 6 week treatment period. Antibiotic treatment after the first 6 weeks will usually be oral, and this is not affected by being in the trial.

To get a reliable result from the trial, we need to include 1050 patients in the study. To help us recruit all the patients we need, several hospitals across the country are taking part.

Why have I been invited?

You are being invited because you have an infection in a bone or joint, which needs treatment with a long course of antibiotics.

Do I have to take part?

No - taking part in a research study is always optional and you do not have to decide straightaway. We will describe the study and go through the information in this leaflet with you. Please feel free discuss the study with friends, family and other patients if you wish. If you agree to take part, we will ask you to sign a consent form before we enrol you into the study. If you later change your mind, you are free to withdraw from the study at any time.

If you choose not to take part, or if you decide to withdraw at any time, this will not affect the standard of care that you receive in your normal treatment.

If you withdraw from receiving the allocated treatment (i.e. intravenous or oral), we would still like to follow your progress for up to a year following your initial involvement with the study.

What happens if I take part?

If you agree to take part, you will be allocated to one of the following types of treatment:

- Oral Antibiotics – these are tablets which you will take between once and four times daily
- Intravenous Antibiotics – these are given as a drip or injection via a narrow plastic tube directly into your vein.

Whichever group you join, your doctor will choose your individual antibiotics according to your specific medical needs and laboratory test results. If you are in the tablet group, you may initially be treated with a short (1 week or less) course of intravenous antibiotics, depending on your type of infection and your ability to swallow tablets.

Both types of treatment will be given for six weeks in the first instance. After the first 6 week treatment period, you may be given further "follow on" antibiotics. This "follow on" antibiotic treatment is not affected by being in the study or by the group you were allocated to for the first 6 week period of treatment.

You will get more detailed information about your antibiotics from the doctor and pharmacist looking after you.

Which group will I be in?

We won't know until we enrol you into the study. Allocation to the groups is determined by a computer so that the process is random like the lottery. Neither you nor your doctor will have a choice as to whether you will receive oral or intravenous antibiotics. We are using this method because it is the best way to allow a fair comparison between the groups. A study that allocates treatment in this way is called a "randomised controlled trial". Whichever group you are in, you will still be under the care of the same clinical care team that have already been treating you.

Will taking part in OvIVA involve extra clinic visits?

No. Any clinic visits or blood tests requested are the same as those needed for your routine care. No additional blood tests or clinic visits will be needed if you join the study and we will not need to store any blood samples.

How often will I be contacted by OvIVA study staff?

We will collect information for the purposes of the trial, either in a routine clinic visit or by telephoning you or your GP, at approximately 6 weeks, 4 months and 12 months after your entry into the study.

What type of information do I need to provide for OvIVA?

At each review we will ask you questions about your progress and any possible side effects or complications of the treatment you have received. If there are signs that the infection is not being successfully treated, we will organise additional tests or treatments that you might need for your clinical care, but we will not ask you to have any procedures or tests that you would not normally require outside the context of the trial.

We will ask you to complete questionnaires about your symptoms and general health when you join the study, and at 2 weeks, 6 weeks, 4 months and 1 year after you start in the study. These questionnaires will be given to you with stamped addressed envelopes when you start the study. You may ask someone to help you complete the questionnaires if you wish.

In order to assess possible difficulties with taking antibiotics, we will be asking patients to fill out a form called a compliance questionnaire. This form will also ask you to estimate how many doses of antibiotics you may have missed and should be filled out as honestly as possible.

Some patients in the 'oral' group will receive their tables in a "MEMS" bottle from pharmacy when they go home. These "MEMS" bottles are designed to record opening and closing of the bottle tops. We will collect the bottles when you return to clinic and record the information, so that we can monitor how regularly tablets were taken. You do not have to use these bottles to participate in the trial if you do not wish to, and the MEMS bottles will only be available in some hospitals. If, despite being treated at one of these hospitals, you prefer not to use one of these MEMS bottles, please inform us and we will provide a standard tablet container.

If you have difficulties taking the medication, or develop symptoms that might be side effects of the antibiotics, or if you notice signs that the infection may be returning, then you must contact us, either through the nurses or doctors running the study or through your GP. A mobile telephone number for a member of the study team is provided in the contact information at the bottom of this form.

What if I forget to take some of my antibiotics?

We know that remembering to take medicines regularly can be difficult but it's important to know that frequent missed doses of antibiotics can lead to the treatment becoming less effective. Therefore, we encourage all of our patients to take their antibiotics as prescribed, even when they are feeling well or think that their infection has been cured.

If you miss a dose, we won't criticise or blame you, and the quality of care that you receive from doctors and nurses will not be affected. However, it is important that we record any missed doses and that you let us know if you are having difficulties for any reason. We may be able to find ways to help, such as changing the times when your antibiotics should be taken, changing the dose or changing to a completely different antibiotic.

A diagram of the steps in the trial is shown below.

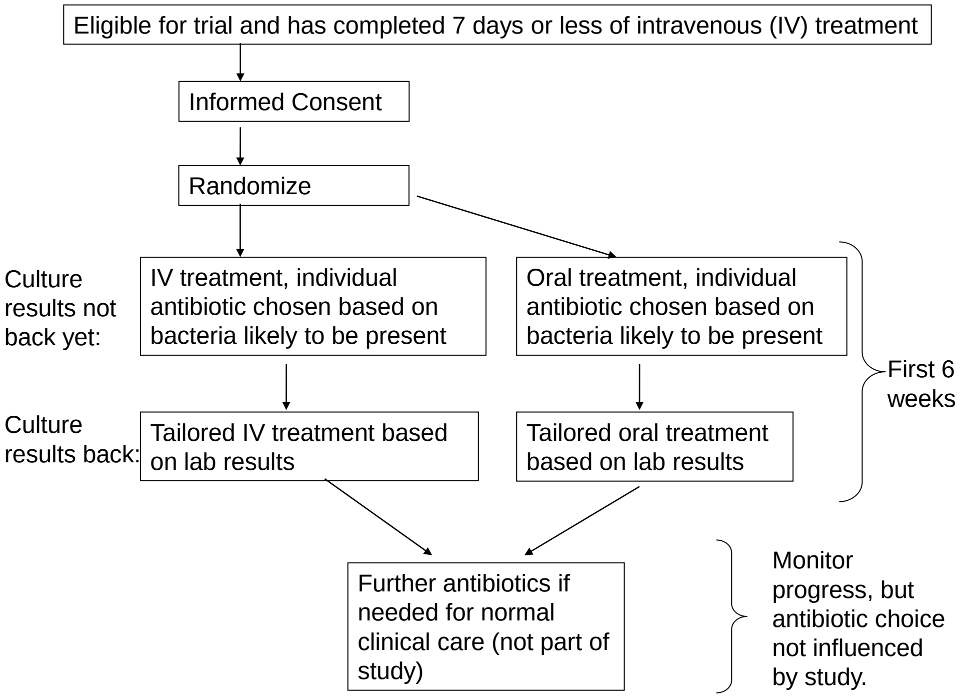

Are there any expenses or payments?

Participating will not involve any extra clinic visits beyond those required for routine clinical care. You will therefore not have any extra expense by taking part and similarly there are no payments for taking part in the study.

What are the possible benefits of taking part?

There are no direct benefits from taking part in the trial other than in helping to identify the best treatment strategy for future patients. However, there are advantages and disadvantages of both intravenous and oral antibiotics.

Intravenous antibiotics are frequently viewed as the standard of care in many hospitals. Patients allocated to the intravenous group are likely to be closely supervised by specialist nurses. However, many patients find intravenous therapy inconvenient. On the other hand, patients allocated to oral therapy may find the tablets easier and more convenient but they are less likely to be closely supervised by nurses and may therefore be at greater risk of missing doses.

What are the possible risks in taking part?

At the moment we do not know which antibiotic strategy (intravenous or oral) is most effective, or whether any difference in effectiveness might be balanced by a difference in side effects. If there is a difference, you might be allocated (by chance) to an

antibiotic strategy that turns out at the end of the trial either to have more side effects or to be less effective.

What are the potential side effects of antibiotic treatment?

The various antibiotics used to treat bone and joint infections all have different side effects, which the doctor looking after you will explain.

In general, antibiotics tablets are more likely to cause nausea and vomiting than antibiotics given by injection. Both tablets and antibiotic injections may cause diarrhoea or a rash.

The initial insertion of an intravenous line used for administering antibiotics by injection involves some discomfort but this is usually mild. Intravenous lines have a small risk of becoming infected themselves and very occasionally, they can cause irritation or blood clots in the vein. Under these circumstances, the line may have to be removed. It is important to remember that risks would be no different if you chose not to be in the trial because it is currently standard practice in your hospital to give antibiotics intravenously in the treatment of bone and joint infection.

If you have any side effects or complications that may be related to an antibiotic or an intravenous line, you must tell the doctors or nurses looking after you.

Might there be harm to an unborn child?

Some antibiotics are not safe for use in pregnancy or breast feeding. If it is possible you might be pregnant, or might become pregnant while taking antibiotics, you must discuss this with your doctor, in order to plan your treatment accordingly. If you are pregnant, it is possible to select both intravenous and oral antibiotics that are of low risk to the unborn child. Antibiotics are not known to damage sperm or affect male fertility, and we do not advise any special precautions for men taking antibiotics.

What alternative treatments are there?

In some centres in the UK, doctors use antibiotic impregnated cement or beads inserted at the time of surgery to treat some infections, without a long course of intravenous or oral antibiotics. However, there are no research studies comparing this treatment option with long courses of antibiotics, and it is not applicable to every type of infection.

What happens if new information becomes available during the trial?

We might receive new information about the risks and benefits of intravenous or oral antibiotic treatment during the course of the study. If this happens, we will inform you of the new information, and discuss whether or not you should continue in the study.

If you do not continue in the study, your doctor will arrange for you to get the required continuing care.

What happens when my involvement in the research stops?

The antibiotic treatment allocation lasts for 6 weeks. At the end of this, the doctor looking after you may prescribe further antibiotics, but this is not influenced by being in the study. The last time at which we record information for the trial will be about one year after you enter the study. Some patients may be kept under follow-up beyond this period independently of the trial; your doctor will inform you if this is necessary.

What happens if I withdraw from the study?

You are free to stop participating at any time after joining the study. However, we will need to use the data collected up to that point in our analysis of the effect of treatment. If you stop receiving the study treatment or if you choose to withdraw from the study then, with your permission, we would still like to continue collecting information about the outcome of your treatment from your medical records, for up to 1 year after you entered the study.

Your doctor may also withdraw you from the study treatment if he/she finds that, because of antibiotic side effects or other factors, you need an antibiotic that isn't available in the oral or intravenous group that you have been allocated.

What happens if I become unable to communicate my wishes to you during the study?

If at any time during the study something happens that results in you being incapacitated or if you become unable to communicate appropriately about the study, we will contact a relative or friend, usually your next of kin. They will be invited to act as your consultee; this is a person who acts in your interests for as long as is necessary in relation to this study. Nobody is forced to be a consultee; if your relative or friend prefers, we will appoint someone else to take on this role.

Your consultee will be given an information sheet very similar to this one so that that they can advise us whether or not we should continue to collect information relevant to the study. If they are happy for us to continue, they will be asked to sign a separate agreement. If they prefer that we don't continue collecting information on your condition, we will respect their wishes. The standard of care that you receive for your medical condition will not be affected by their decision.

What if there is a problem?

If you have a complaint, or suspect you have suffered harm as a result of the study, we will address this as described in Part 2. If the information in Part 1 has interested you, please read part 2 before making a decision.

Part 2: Confidentiality, study results and what to do if things go wrong.

Will my information be kept confidential?

Yes. We follow the legal and ethical standards that ensure this. Any information about you that is stored electronically will be identified only by your NHS number and a unique study number. Your name and other personal details will be linked to your study number on a separate paper record which will be stored securely in a locked filling cabinet.

We will tell your GP that you are participating in the trial, and supply them with our contact details in case they have any concerns. We may also telephone your GP to find out about your progress.

If we identify important clinical information about you during the study, we will need to ensure that this is contained in your hospital file that other doctors use. If necessary for your clinical care, we will inform other doctors or nurses about this information.

Study monitors may need to review your hospital records in order to ensure that the information we collect is accurate but they will not remove your case file from the hospital.

We will keep the electronic data for 20 years after the end of the trial. It is possible that this data will at some stage be used for further ethically approved studies of antibiotic treatment. Data collected during OvIVA may be shared with other researchers outside of the European Union where laws may not protect the privacy of data to the same extent as they do in the UK. However, none of the data stored or transferred electronically for this purpose will contain personal identifiers other than your study number, so it should not be possible to identify any individual patients .

What happens to the results of the trial?

A summary of the results may be presented at scientific meetings, or published in journals. In these presentations, no information that would identify an individual participant will be presented.

Who is organising and funding the research?

The study has been designed by a group of doctors working in infectious disease and surgery in Oxford. These doctors receive salary support from the NHS and from Oxford University. Funding for the trial comes from the National Institute of Health Research. The study is sponsored by Oxford University Hospitals NHS Trust Research and Development department.

Who has reviewed the study?

To protect your interests, all research in the NHS is scrutinised by an independent group of people, called a Research Ethics Committee. The reference number allocated by the committee which reviewed this study is shown at the top of each page of this information sheet.

What if there is a problem?

The NHS hospital remains responsible for your care during the study. If you have a concern about any aspect of the study, you should speak to the study doctor or a member of the research team for information. If you wish to proceed formally with a complaint, this can be done through the NHS complaints procedure, most usually through the Patient Advice and Liaison Service

If something goes wrong and you suffer harm during the research due to someone's negligence, then you may have grounds for a legal action for compensation but you may have to pay your legal costs.

Contact information

There is a 24 hour service run from ...……...……. Hospital which a GP or out of hours doctor can be contacted in a clinical emergency. You may also have been given contact numbers for specialist nursing teams who you can contact with any questions or concerns. The Trial Nurse can be contacted on………......………..

If you wish to discuss any aspect of the study with a doctor, you can contact your local study doctor in the If you wish to speak to the central study co-ordinator in Oxford you can ring.............................

If you have a concern regarding the study, and want to contact someone independent, you can contact the Patient Advice and Liaison Service on……....……….
