# Supplementary material for: Oral versus intravenous antibiotic treatment for bone and joint infections (OVIVA): study protocol for a randomised controlled trial
Source: Trials. 2015 Dec 21;16:583. doi: 10.1186/s13063-015-1098-y (PMC4687165; doi:10.1186/s13063-015-1098-y)
Supplement: Additional file 2: — OVIVA Consent Form. Form used in the OVIVA trial to record informed consent. (PDF 13 kb) [file 13063_2015_1098_MOESM2_ESM.pdf]

CONSENT FORM ***Participant Study Number***

Study Title: Randomized open label study of oral versus intravenous antibiotic treatment for bone and joint infections requiring prolonged antibiotic treatment: Multi-Centre Study

1. I confirm that I have read and understood the information sheet dated _____, version ____ for the above study, I have had the opportunity to ask questions and these have been answered to my satisfaction. ☐

2. I understand that my participation is voluntary and that I am free to withdraw at any time, without giving any reason, without my medical care or legal rights being affected. ☐

3. If I become incapacitated for any reason during the trial, I am happy for the trial staff to continue collecting information relevant to the study for up to one year, provided that a relative, friend or guardian signs a separate agreement to this as a consultee ☐

4. I understand that relevant sections of my medical notes and data collected during the study may be looked at by individuals from Oxford University Hospitals NHS trust, from regulatory authorities or from the NHS Trust, where it is relevant to my taking part in this research. I give permission for these individuals to have access to my records. ☐

5. I agree that my next of kin, my GP and other medically qualified people who may assist in my care during the study may be notified of my participation in the study. ☐

6. I agree that medically important information identified in the study may be disclosed to my GP and other medically qualified people involved in my care. ☐

7. I agree that data from my participation may be used in further ethically approved studies of antibiotic treatment. This may include use of my data outside the European Union where laws may not protect data privacy to the same extent as in the UK. To ensure confidentiality, none of the data stored or transferred electronically contains personal identifiers. ☐

8. I agree that data from samples gathered as part of my routine medical care may be used for research purposes. ☐

9. If I have been given a MEMS bottle which records when I take medication, then I agree that the records from this bottle can be used in the study or by medically qualified people involved in my care. ☐

10. I agree to take part in the study. ☐

____________________	____________________	________________
Name of Patient	Signature	Date
____________________	____________________	________________
Name of Person taking consent<br>(if different from researcher)	Signature	Date
____________________	____________________	________________
Researcher	Signature	Date

1 for patient; 1 for researcher; 1 to be kept with hospital notes
